# Association between triglyceride glucose index and risk of acute kidney injury in critically ill patients: a systematic review and meta-analysis

**DOI:** 10.3389/fendo.2026.1759218

**Published:** 2026-02-26

**Authors:** Maoying Wei, Kaixuan Chu, Chan Wu, Haoshuo Wang, Aijing Li, Jingyi Guo, Anning Sun, Xin Gu, Yuyun Fan, Zhijuan Tan, Yanbing Gong

**Affiliations:** 1 Dongzhimen Hospital, Beijing University of Chinese Medicine, Beijing, China; 2 Department of Traditional Chinese Internal Medicine, Linyi People’s Hospital, Linyi, China; 3 Department of Traditional Chinese Medicine, Xingtai Hospital of Traditional Chinese Medicine, Xingtai, Heibei, China

**Keywords:** acute kidney injury, insulin resistance, meta-analysis, systematic review, triglyceride glucose index

## Abstract

**Background:**

Acute kidney injury (AKI) is a common and serious complication among critically ill patients. The triglyceride-glucose (TyG) index, a simple surrogate marker of insulin resistance (IR), has recently emerged as a potential predictor of AKI in this population. However, the existing evidence has not yet been systematically evaluated.

**Objective:**

To systematically evaluate the association between the TyG index and the risk of AKI in critically ill patients.

**Methods:**

A comprehensive literature search was performed across PubMed, Embase, and Web of Science from inception to October 31, 2025, for observational studies reporting the relationship of the TyG index with AKI risk among critically ill patients. Following predefined eligibility criteria, two authors independently undertook the screening process, data extraction using a standardized data collection form, and risk of bias evaluation. All statistical analyses were carried out with RevMan 5.3 and Stata 16.0.

**Results:**

A total of 18 studies involving 81,479 participants were included in the meta-analysis. The results demonstrated that a higher TyG index was significantly associated with an increased risk of AKI, with a pooled odds ratio (OR) of 1.39 (95% CI: 1.22-1.58, *P* < 0.00001) and a pooled hazard ratio (HR) of 1.43 (95% CI: 1.14-1.78, *P* = 0.002). This positive association remained consistent across most subgroups stratified by factors such as sex, age, hypertension, chronic kidney disease, and diabetes. However, the association did not reach statistical significance in the Black population or in subgroups with or without atrial fibrillation.

**Conclusion:**

The TyG index was significantly associated with the risk of AKI in critically ill patients, with higher TyG index levels correlating with an increased risk of AKI.

**Systematic Review Registration:**

https://www.crd.york.ac.uk/prospero, identifier CRD420251232658.

## Introduction

Acute kidney injury (AKI) is a clinical syndrome characterized by a rapid decline in renal function over a short period, resulting from various etiologies. It is primarily manifested by a decrease in glomerular filtration rate and the accumulation of nitrogenous waste products, such as creatinine and urea, in the body. AKI is particularly common among critically ill patients and is often associated with a poor prognosis. It has been reported that the incidence rate of AKI in intensive care unit patients can exceed 50%, which is significantly higher than that in general hospitalized patients (approximately 10%-15%) (1). AKI is not only closely associated with short-term outcomes such as prolonged hospital stays, increased healthcare costs, and elevated in-hospital mortality, but also serves as a predictor for long-term risks including chronic kidney disease, renal failure, and cardiovascular events (2–4). Therefore, early identification of high-risk AKI patients and implementation of interventions in critically ill populations are of great importance for improving clinical outcomes.

Currently, the diagnosis of AKI primarily relies on serum creatinine levels and changes in urine output. However, serum creatinine levels are influenced by various non-renal factors and exhibit a significant lag after the onset of kidney injury, which limits their effectiveness for early risk prediction (5–7). In recent years, researchers have increasingly recognized the central role of metabolic disturbances in the development and progression of AKI. In critically ill patients, insulin levels are elevated while insulin sensitivity is reduced (8). As a key pathophysiological condition, insulin resistance (IR) directly contributes to the progression of renal injury through multiple mechanisms, including reduced nitric oxide production in endothelial cells, increased oxidative stress, and the promotion of inflammation (9, 10). It was found by Ekperikpe et al. (11) that metformin’s ability to reduce insulin resistance can prevent pre-pubertal renal hyperfiltration and progressive renal injury in SSLepR mutant rats. Another report indicated that improving insulin resistance by increasing renal INSR and eNOS expression also helps mitigate obesity-induced podocyte injury in mice (12).

Current methods for assessing insulin resistance include the hyperinsulinemic-euglycemic clamp, the minimal model method from the intravenous glucose tolerance test, the homeostasis model assessment of insulin resistance (HOMA-IR), the quantitative insulin sensitivity check index, and the Matsuda index (13, 14). The triglyceride-glucose (TyG) index has emerged as a novel, simple, and reliable surrogate marker of insulin resistance (IR). Its calculation requires only fasting triglyceride and fasting blood glucose levels, thereby circumventing the complexity of the hyperinsulinemic-euglycemic clamp technique (the gold standard) and the limitations of the HOMA-IR index (15–18). Studies have demonstrated that the TyG index is significantly associated with the risk of cardiovascular and renal diseases in the general population (19, 20). In the field of critical care, the TyG index has been shown to correlate with mortality in various severe conditions, including ischemic stroke, sepsis, hemorrhagic stroke, and atrial fibrillation (21–24). It is noteworthy that recent observational studies have begun to explore the association between the TyG index and the risk of AKI in critically ill patients, with preliminary results suggesting its potential predictive value (25–27).

However, the existing evidence has not yet reached a consistent conclusion. Studies vary in sample size, population characteristics, and effect sizes, with limited statistical power in individual studies. To date, no research has systematically synthesized and quantitatively analyzed this specific association. Therefore, this study aims to systematically integrate existing observational evidence, evaluating the association between a high TyG index and AKI risk in critically ill patients, and to provide higher-level evidence for early clinical risk stratification.

## Methods

The research adhered to the Preferred Reporting Items for Systematic Reviews and Meta-Analyses (PRISMA) 2020 statement **Supplementary Table 1** (28). The study protocol is publicly available in the PROSPERO database under registration number CRD420251232658.

### Search strategy

A comprehensive literature search was conducted in PubMed, Embase, and Web of Science databases from their inception until October 31, 2025, to identify studies exploring the relationship between the TyG index and the risk of AKI. The search strategy combined subject headings (e.g., MeSH) with free-text terms, including “triglyceride glucose index, or triglyceride-glucose index, or TyG index”, and “acute kidney injury, or acute renal injury, or acute kidney injuries”, and “critical illness, or critical patients, or critically ill patients, or critical care”. Additionally, the reference lists of included articles were manually screened to supplement the search. The detailed search strategy is provided in **Supplementary Table 2**.

### Inclusion criteria

(1) Type of studies: Both prospective and retrospective observational studies were included in the meta-analysis. (2) Type of participants: Critically ill patients, irrespective of disease, race, nationality, age, or gender. (3) Exposure factor: Individuals with a high TyG index constituted the exposure group, while those with a low TyG index served as the control group. The TyG index is calculated based on fasting blood glucose and triglycerides measured upon admission, using the formula: ln[TG (mg/dL) × FBG (mg/dL)/2] (15). (4) Outcomes: The risk of AKI. Acute kidney injury (AKI) was defined according to the Kidney Disease: Improving Global Outcomes (KDIGO) criteria, which requires an increase in serum creatinine by ≥ 0.3 mg/dL (≥ 26.5 μmol/L) within 48 hours, or an increase to ≥ 1.5 times the baseline value within 7 days, or a urine output of < 0.5 mL/kg/h for more than 6 hours (29).

### Exclusion criteria

(1) Non-English publications; (2) Duplicate publications or studies with unavailable full text; (3) Studies with inaccessible or irretrievable original data; (4) Conference abstracts, case reports, reviews, letters, and commentaries.

### Literature screening and data extraction

Literature screening and data extraction were performed independently by two reviewers (M.W. and K.C.). Duplicate records were removed using EndNote (version X9.1, Clarivate Analytics, Philadelphia, PA, USA), followed by a manual check. The unique records were then independently screened (titles/abstracts, then full-texts) and managed using a shared Excel spreadsheet. Inter-reviewer agreement was quantified as percentage agreement (92% at title/abstract stage; 90% at full-text stage). Data were extracted using a piloted form. All disagreements were resolved by consensus or, if needed, by arbitration from a third reviewer (Y.F.). The extracted information included: (1) basic characteristics of the included studies, such as the first author, year of publication, study design, data source, and sample size; (2) baseline characteristics of the study participants, including age, gender, and disease status; (3) key elements for assessing the risk of bias; and (4) outcome measures and corresponding data. In cases where multiple models were presented in the original studies (e.g., in multivariate analyses), the model that was most fully adjusted for potential confounders was selected. The TyG index can be included in data extraction and synthesis both as a continuous variable and as a categorical variable. If an original study evaluated the TyG index as a categorical variable, the estimate for the highest quartile relative to the first quartile should be extracted. If the TyG index was assessed as a continuous variable, the estimate per unit increase should be extracted.

### Quality assessment

Two reviewers (M.W. and A.S.) independently evaluated the risk of bias for all included studies. The Newcastle-Ottawa Scale (NOS) was used for cohort studies, with scoring conducted per its standard guidelines (30). Studies were categorized as high (>7 points), moderate (5–7 points), or low (0–4 points) quality. Cross-sectional studies were assessed using the Agency for Healthcare Research and Quality (AHRQ) tool (11 items), and classified as high (8–11 points), moderate (4–7 points), or low (0–3 points) quality (31). Inter-rater reliability was assessed by calculating Cohen’s kappa (κ) for the initial quality classifications, yielding a value of 0.64 (substantial agreement). Any scoring discrepancies were resolved through consensus discussion between the two reviewers, with unresolved cases adjudicated by a third senior reviewer (Y.F.).

### Statistical analysis

Statistical analyses were performed using Review Manager software (version 5.3, Copenhagen: The Nordic Cochrane Center, The Cochrane Collaboration, 2014) and Stata software (version 16, The Stata Corporation, College Station, Texas, USA). Studies reported the association between the TyG index and AKI risk using different adjusted effect measures, primarily as hazard ratios (HRs) or odds ratios (ORs). Given the distinct methodological foundations and interpretations of these measures, no statistical conversions were performed between HRs and ORs, and they were not combined in a single meta-analysis. The effect measures were expressed as ORs or HRs, with results presented as point estimates and their 95% confidence intervals (CIs). Prior to data pooling, logarithmic transformations were applied to the ORs, HRs, and 95% CIs to approximate a normal distribution. We assessed statistical heterogeneity across studies with the I² statistic. An I² value ≤ 50% indicated the use of a fixed-effects model, while an I² > 50% prompted the use of a random-effects model and further investigation into heterogeneity sources via subgroup or sensitivity analyses. We examined publication bias with funnel plots and Egger’s test, setting a significance threshold at *P* < 0.05.

## Results

### Literature screening process and results

**Figure 1:**
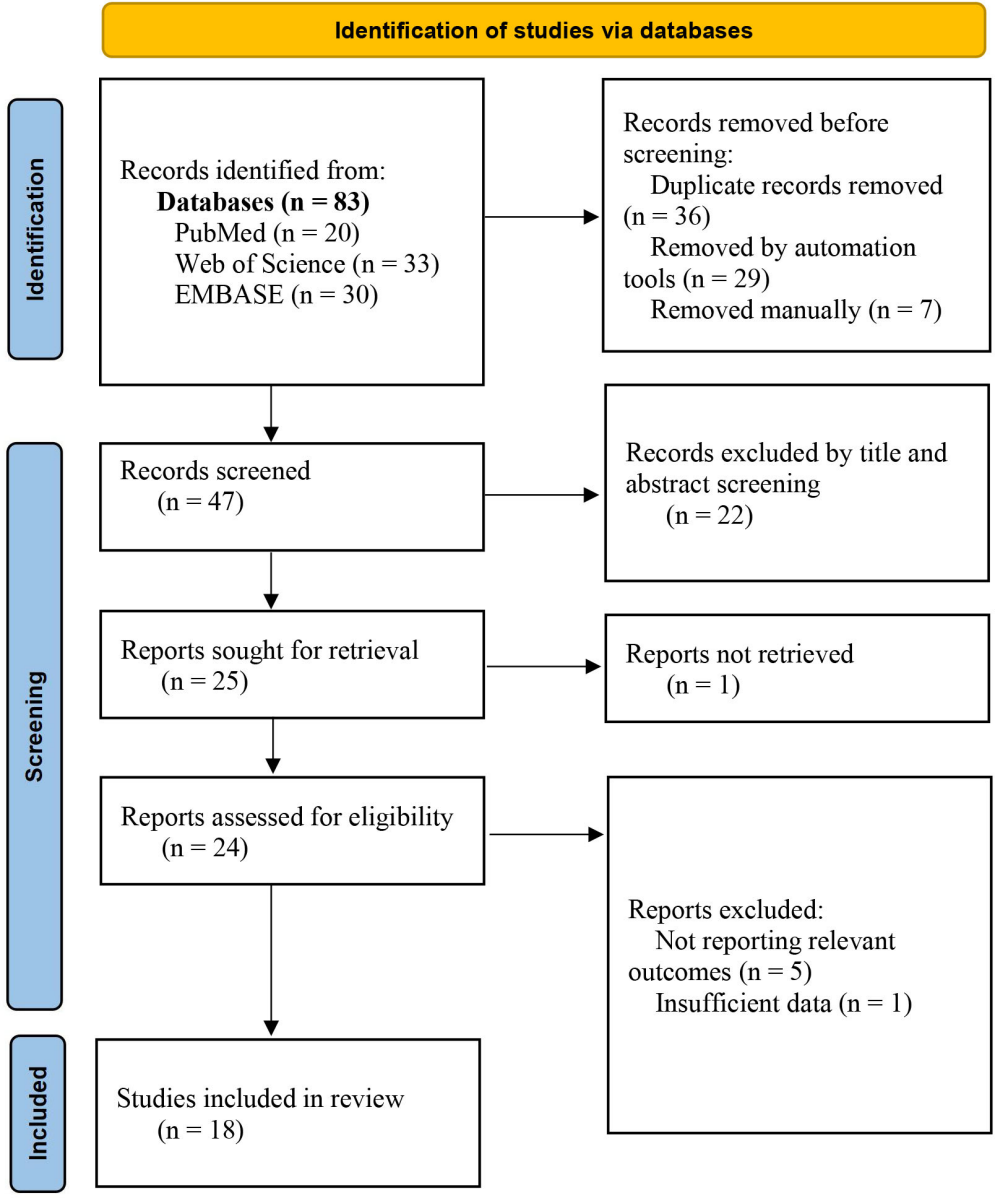
Flowchart of the study selection process.

A total of 83 relevant articles were retrieved in this study, including 20 from PubMed, 33 from Web of Science, and 30 from EMBASE. After a layer-by-layer screening process, 18 studies were ultimately included, comprising 15 retrospective cohort studies and 3 cross-sectional studies. The literature screening process and results are shown in **Figure 1**.

### Basic characteristics and quality assessment results of the included studies

The included studies were published between 2023 and 2025. Data from 13 studies (25, 27, 32–42) were sourced from the Medical Information Mart for Intensive Care (MIMIC) database, one study (43) from the eICU Collaborative Research Database (eICU-CRD), and four studies (44–47) were based on retrospective cohort studies conducted at four tertiary hospitals in China. The total sample size was 75,199 participants, with individual study sample sizes ranging from 435 to 54,263. The critical illnesses encompassed in the patient cohorts included acute myocardial infarction, sepsis, severe traumatic brain injury, atrial fibrillation, acute pancreatitis, aneurysmal subarachnoid hemorrhage, heart failure, and hypertension, among others. The risk of bias assessment indicated that two studies (34, 35) were of moderate quality (AHRQ scores of 6-7), while sixteen studies (25, 27, 32, 33, 36–47) were of high quality (one with an AHRQ score of 8, and fifteen with NOS scores of 8). The detailed baseline characteristics and quality assessment of the included studies are presented in **Table 1**.

**Table 1:** Basic characteristics of included studies.

Study ID	Study type	Database	Sample size	Age	Male (%)	Diseases	Subgroup analysis	Endpoint	TyG analysis type	AHRQ/NOS
Cai DB 2024	Cohort study	MIMIC v1.4 MIMIC-IV	1831	18-90 year	68.9	AMI	Gender, age, AF, hypertension, DM	AKI	Contimuous	8
Fang YJ 2024	Cohort study	MIMIC-IV	1426	62.1±17.5	56.4	Sepsis	Age, gender, DM, CKD	AKI	Contimuous	8
Hou B 2025	Cohort study	Beijing Anzhen Hospital	3260	64.0(57.0,69.0)	74.2	coronary artery bypass grafting	Age, gender, BMI, hypertension, DM	AKI	Contimuous	8
Huang J 2024	Cross-sectional study	MIMIC-IV	492	68.9±15.9	55.9	Severe traumatic brain injury	Age, gender, race, DM	AKI	Contimuous	6
Jin ZH 2023	Cross-sectional study	MIMIC-IV	54263	59.3±17.7	48.2	AMI, AF, cerebral infarction, etc.	Age, AF	AKI	Contimuous	7
Jin ZH 2024	Cross-sectional study	MIMIC-IV	1101	64.5±12.9	64.7	AMI	Gender, hypertension	AKI	Contimuous	8
Lu Y 2025	Cohort study	MIMIC-IV	1142	71.7±13.16	55.3	Critical AF	Age, gender, race, hypertension, DM, CKD	AKI	Contimuous	7
Pan RJ 2025	Cohort study	Fujian Provincial Hospital	1505	55.47±17.32	66.45	traumatic brain injury	Age, gender, hypertension, DM	AKI	Contimuous	8
Qiu XY 2025	Cohort study	West China Hospital	3271	55.11±11.93	34.9	aneurysmal subarachnoid hemorrhage	Age, gender, hypertension, DM	AKI	Contimuous	8
Shi Y 2024	Cohort study	MIMIC-IV	790	67.65(58.59,76.99)	71.8	Coronary revascularization	Age, gender, BMI, hypertension, DM, CKD	AKI	Contimuous	8
Wang X 2024	Cohort study	eICU-CRD	645	67.4±10.9	48.5	AECOPD	Age, gender, race, hypertension, DM, CKD	AKI	Contimuous	8
Wang Z 2025	Cohort study	MIMIC-IV	848	56.89±16.76	58.6	Acute pancreatitis	Age, gender, BMI, hypertension, DM, CKD	AKI	Contimuous	8
Yang ZW 2023	Cohort study	MIMIC-IV	1393	71(60,81)	59.0	Heart failure	Age, gender, AMI, hypertension, DM, CKD, BMI	AKI	Contimuous	8
Zhang F 2025	Cohort study	MIMIC-IV	435	67.14±13.72	66.9	Patients undergoing percutaneous coronary	Age, gender, hypertension, DM, BMI	AKI	Contimuous	8
Zhang PR 2025	Cohort study	MIMIC-IV	2616	–	57.0	Sepsis	Gender, race, AF, hypertension, DM, CHF, BMI	AKI	Contimuous	8
Zhang WB 2024	Cohort study	MIMIC-IV	4418	67(57,78)	56.6	Critical hypertension	Gender, CKD, AMI, age, DM, BMI	AKI	Contimuous	8
Zhang Y 2025	Cohort study	MIMIC-IV	1501	69.80 (59.72,80.45)	61.6	Coronary artery disease	Gender, age, hypertension, CHF, CKD, DM	AKI	Contimuous	8
Zhang Z 2025	Cohort study	Zhongshan Hospital, Fudan University	542	–	59.6	Patients with chronic kidney disease undergoing cardiac surgery	Age, gender, BMI, hypertension, DM	AKI	Contimuous	8

AECOPD, acute exacerbation of chronic obstructive pulmonary disease; AF, atrial fibrillation; AHRQ, Agency for Healthcare Research and Quality; AKI, acute kidney injury; AMI, acute myocardial infarction; BMI, Body mass index; CHF, congestive heart failure; CKD, chronic kidney disease; DM, diabetes mellitus; eICU-CRD, the eICU Collaborative Research Database; MIMIC, Medical Information Mart for Intensive Care; NOS, Newcastle-Ottawa Scale; PIC, Pediatric Intensive Care; TyG, triglyceride glucose index.

### Quantitative analysis

**Figure 2:**
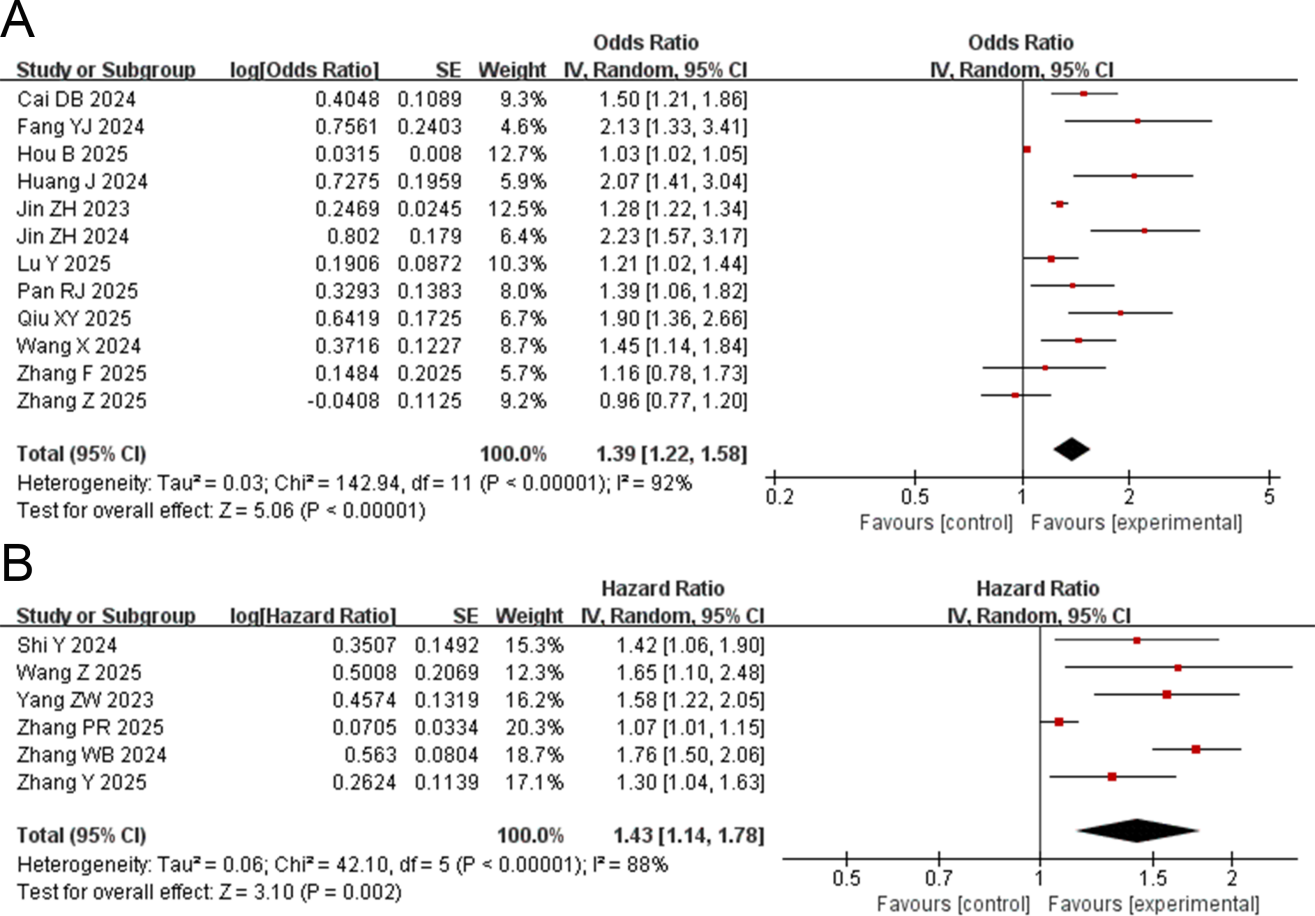
Forest plot for the association between triglyceride glucose index and risk of acute kidney injury in critically ill patients. **(A)** odds ratio (OR) and **(B)** hazard ratio (HR).

Twelve studies (32–37, 39, 43–47) evaluated the association between the TyG index and the risk of AKI using OR. The pooled results demonstrated that the TyG index is a risk factor for AKI (OR = 1.39, 95% CI: 1.22-1.58, *P* < 0.00001) (**Figure 2A**). Subgroup analyses were conducted based on study design (cohort or cross-sectional), sex (male or female), age (older or younger), chronic kidney disease (with or without chronic kidney disease), hypertension (with or without hypertension), and diabetes mellitus (with or without diabetes mellitus). The results indicated that a high TyG index was significantly associated with the occurrence of AKI in all these subgroups. However, subgroup analyses revealed no significant association between a high TyG index and AKI risk in several strata: the high BMI group (OR = 1.35, 95% CI: 0.95-1.90, *P* = 0.09), Black participants (OR = 1.36, 95% CI: 0.92-2.00, *P* = 0.12), patients with atrial fibrillation (OR = 1.45, 95% CI: 0.92-2.29, *P* = 0.11), and those without atrial fibrillation (OR = 1.68, 95% CI: 0.95-2.98, *P* = 0.07) (**Supplementary Table 3**, **Supplementary Figure 1**).

Six studies (25–27, 38, 40, 42) reported the association between the TyG index and the risk of AKI in the form of HRs. The pooled results demonstrated that individuals with a high TyG index had a significantly higher risk of AKI compared to those with a low TyG index (HR = 1.43, 95% CI: 1.14-1.78, *P* = 0.002) (**Figure 2B**). Subgroup analyses based on gender (male or female), age (older or younger), body mass index (higher or lower), chronic kidney disease (with or without chronic kidney disease), hypertension (with or without hypertension), diabetes mellitus (with or without diabetes mellitus), congestive heart failure (with or without congestive heart failure), and acute myocardial infarction (with or without acute myocardial infarction) indicated that a high TyG index remained a risk factor for AKI across all subgroups (**Supplementary Table 4**, **Supplementary Figure 2**).

### Sensitivity analysis

**Figure 3:**
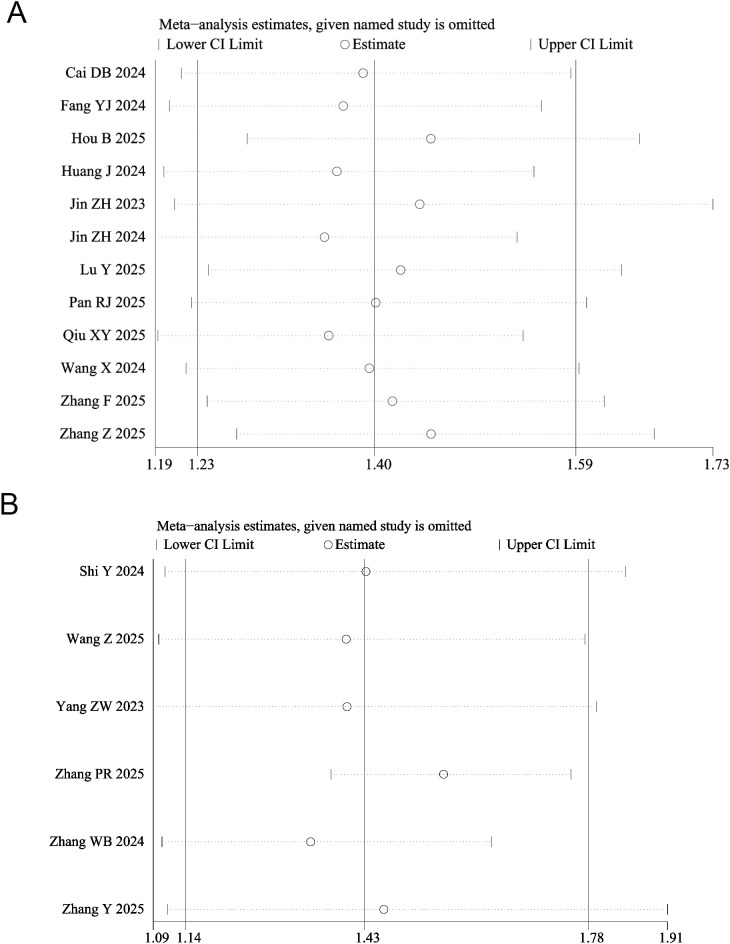
Sensitivity analysis for the association between triglyceride glucose index and risk of acute kidney injury in critically ill patients. **(A)** odds ratio (OR) and **(B)** hazard ratio (HR).

The sensitivity analysis revealed that after excluding any single study, the recalculated pooled ORs ranged from 1.23 to 1.59, showing no significant deviation from the original pooled estimate (OR = 1.39, 95% CI: 1.22-1.58) based on all included studies (**Figure 3A**; **Supplementary Table 5**). Similarly, the point estimates of the pooled HR remained stable, ranging from 1.14 to 1.78, with confidence intervals broadly overlapping those of the original pooled result (HR = 1.43, 95% CI: 1.14–1.78) (**Figure 3B**; **Supplementary Table 5**). These findings indicate that the results of this meta-analysis are highly robust and that the conclusions are not unduly influenced by any individual study.

### Publication bias

**Figure 4:**
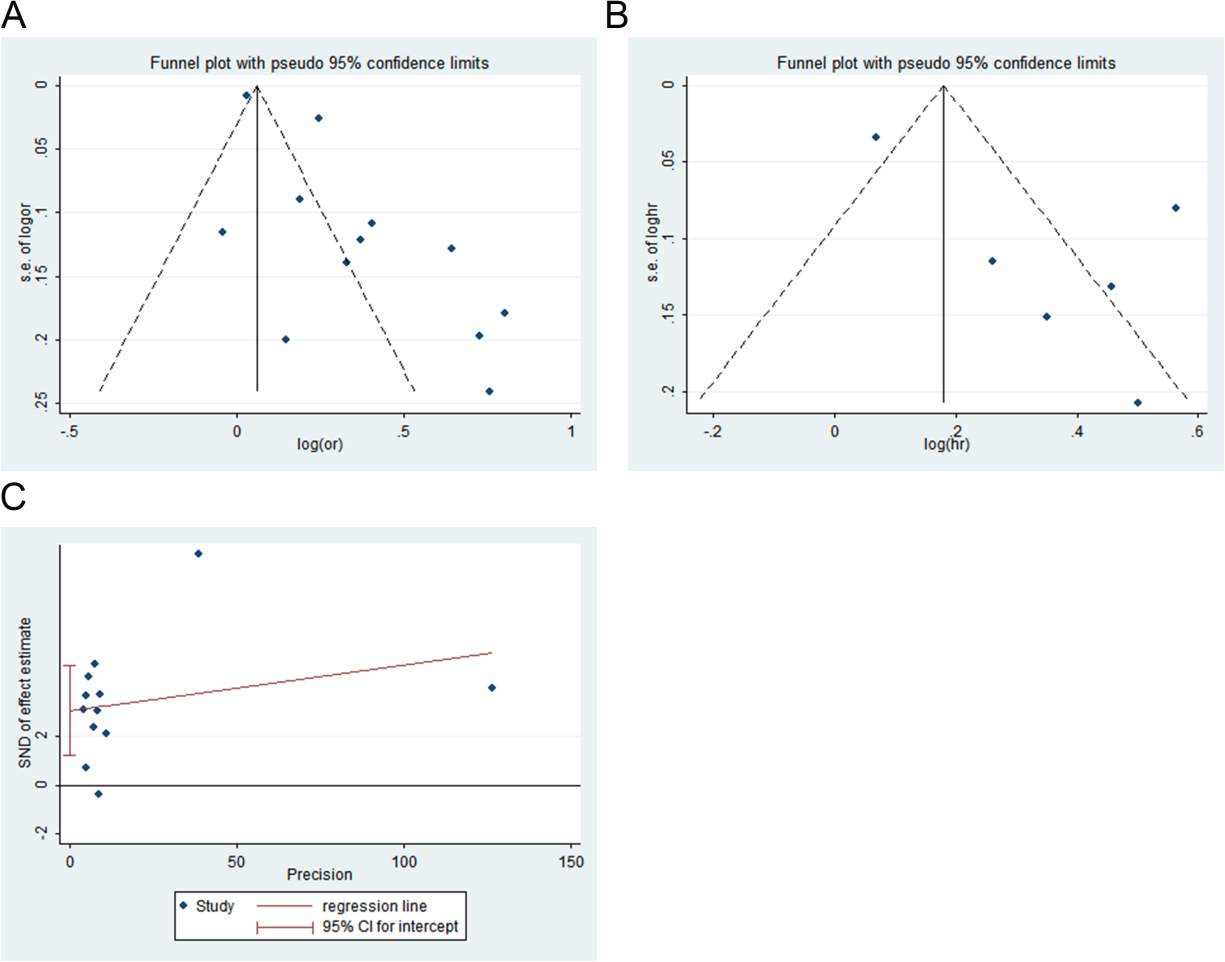
Publication bias for the association between triglyceride glucose index and risk of acute kidney injury in critically ill patients. **(A)** funnel plots of 0dds ratio (OR), **(B)** funnel plots of hazard ratio (HR), and **(C)** Egger’s test of OR.

Publication bias among the included studies was first assessed qualitatively using funnel plots. Visual inspection revealed asymmetry in the funnel plot (**Figures 4A, B**). In accordance with methodological recommendations, a quantitative assessment using Egger’s linear regression test was performed only for outcomes with 10 or more included studies. The result indicated statistically significant publication bias (t = 3.71, P = 0.04; **Figure 4C**).

## Discussion

### Summary of results

This study, through a systematic review and meta-analysis, provides the first comprehensive assessment of the association between the TyG index and the risk of AKI in critically ill patients. The results indicate that a high TyG index is an independent risk factor for AKI, with a pooled OR of 1.39 (95% CI: 1.22-1.58) and a pooled HR of 1.43 (95% CI: 1.14-1.78). These findings confirm that the TyG index is not only significantly associated with AKI in cross-sectional studies but also effectively predicts the long-term risk of AKI in cohort studies, providing robust evidence-based support for its use as an early indicator of AKI in critically ill patients.

IR is a pathophysiological condition in which target organs of insulin action (primarily the liver, skeletal muscle, and adipose tissue) exhibit reduced sensitivity to insulin. This impairment leads to ineffective promotion of glucose uptake and utilization, thereby triggering a series of metabolic disorders. Studies have found that obesity-induced IR rat models exhibit significant renal dysfunction, characterized by elevated serum creatinine levels and increased microalbuminuria, along with exacerbated oxidative stress and inflammatory responses (48). Yu et al. (49) reported that Akt2 gene deficiency-induced IR mice not only exhibited systemic glucose metabolic disorders but also showed significant renal tissue oxidative damage, apoptosis, and excessive autophagy. Other studies have indicated that IR can promote the onset and progression of diabetic nephropathy independently of blood glucose levels. For instance, knockout of the TRPC6 gene in Akita mouse models further exacerbates IR and aggravates glomerulopathy independently of hyperglycemia (50). Therefore, IR is considered one of the critical factors contributing to renal injury.

The TyG index was first introduced by Simental-Mendía et al. (15) in 2008. Previous studies have shown that the TyG index performs comparably or even superiorly to HOMA-IR in assessing IR (51, 52). Currently, this index has been widely applied in research related to various kidney diseases, including chronic kidney disease, diabetic nephropathy, kidney stones, and acute renal failure (20, 53–56). In the field of AKI research, a higher TyG index is significantly associated with the incidence of delirium, short-term mortality, and all-cause mortality in critically ill AKI patients (57–59). Studies have also confirmed that the preoperative TyG index serves as an important independent predictor of AKI following coronary artery bypass grafting (44). Li et al. (60) reported that for each unit increase in the TyG index, the risk of AKI after coronary artery bypass grafting increased by 30.573 times (OR = 30.573, 95% CI: 3.930-237.807, *P* < 0.001). Furthermore, the TyG index has also been identified as an independent predictor of AKI and mortality in patients with aneurysmal subarachnoid hemorrhage (46). Pan et al. (45) also observed a significant positive correlation between TyG levels and AKI in patients with traumatic brain injury. Consistent with previous findings, our study similarly demonstrated a positive association between the TyG index and the risk of AKI, further supporting the potential value of the TyG index in assessing AKI risk.

Subgroup analysis further revealed the stability of this association across different populations. In the vast majority of clinically relevant subgroups (such as those stratified by sex, age, and the presence or absence of chronic kidney disease, hypertension, diabetes, etc.), a high TyG index was consistently associated with an increased risk of AKI, indicating that this association is relatively generalizable across critically ill patients with different characteristics. However, no statistically significant association was observed in the following subgroups: those with high body mass index, Black participants, and patients with or without atrial fibrillation. This may be attributed to the relatively small sample sizes in these subgroups, metabolic differences across ethnicities, or confounding effects related to hemodynamic disturbances and medications associated with atrial fibrillation itself. These findings suggest that future studies should more specifically explore these particular populations to clarify the boundary conditions for the application of the TyG index.

### Strength and limitation

The strengths of this study include: a comprehensive search of three major databases, the inclusion of all recently published relevant studies, and a relatively large total sample size. Strict literature quality assessment criteria were applied, resulting in an overall high quality of the included studies. Both ORs and HRs were pooled, validating the strength and consistency of the associations from different statistical perspectives. Furthermore, thorough subgroup analyses and sensitivity analyses confirmed the robustness of the findings across various clinical scenarios.

This study has several limitations. First, the included studies were observational in design; thus, while they indicate an association, causality cannot be established, and residual confounding cannot be fully ruled out. Second, funnel plot asymmetry and Egger’s test suggested potential publication bias, indicating that unpublished small studies or negative findings might have been missed, possibly leading to an overestimation of the effect size. Third, although subgroup analyses were performed, the association lost statistical significance in populations with high BMI, Black individuals, and those with atrial fibrillation, implying the presence of unmeasured confounding factors or population-specific heterogeneity. Finally, the majority of the studies (13 out of 18) were predominantly derived from the MIMIC database, raising concerns regarding potential data overlap and limited generalizability, as the population is primarily from Western countries.

## Conclusion

This study demonstrates that a higher TyG index is significantly associated with an increased risk of AKI in critically ill patients. As a simple, economical, and readily accessible parameter, the TyG index offers a promising tool for early identification and risk stratification of AKI in this population.

## Data Availability

The original contributions presented in the study are included in the article/**Supplementary Material**. Further inquiries can be directed to the corresponding authors.
