# Supplementary material for: Association between triglyceride glucose index and risk of acute kidney injury in critically ill patients: a systematic review and meta-analysis
Source: Front Endocrinol (Lausanne). 2026 Feb 26;17:1759218. doi: 10.3389/fendo.2026.1759218 (PMC12979098; doi:10.3389/fendo.2026.1759218)

**Association between triglyceride glucose index and risk of acute kidney injury in critically ill patients: A systematic review and meta-analysis Supplementary Files**

[Supplementary Table 1: PRISMA 2020 Checklist 1](#_Toc206786638)

[Supplementary Table 2 Systematic search detail (record number: 2025.10.31) 6](#_Toc206786639)

[Supplementary Table 3 Results of subgroup analysis (OR) 9](#_Toc206786641)

[Supplementary Table 4 Results of subgroup analysis (HR) 1](#_Toc206786641)1

[Supplementary Table 5 Sensitivity analysis 1](#_Toc206786641)2

[Supplementary Fig. 1 Subgroup analysis of AKI (OR) 1](#_Toc206786643)3

[Supplementary Fig. 2 Subgroup analysis of AKI (HR) 1](#_Toc206786644)7

# Supplementary Table 1: PRISMA 2020 Checklist

| **Section and Topic** | **Item #** | **Checklist item** | **Location where item is reported** |
| --- | --- | --- | --- |
| **TITLE** | | |  |
| Title | 1 | Identify the report as a systematic review. |  |
| **ABSTRACT** | | |  |
| Abstract | 2 | See the PRISMA 2020 for Abstracts checklist. |  |
| **INTRODUCTION** | | |  |
| Rationale | 3 | Describe the rationale for the review in the context of existing knowledge. |  |
| Objectives | 4 | Provide an explicit statement of the objective(s) or question(s) the review addresses. |  |
| **METHODS** | | |  |
| Eligibility criteria | 5 | Specify the inclusion and exclusion criteria for the review and how studies were grouped for the syntheses. |  |
| Information sources | 6 | Specify all databases, registers, websites, organisations, reference lists and other sources searched or consulted to identify studies. Specify the date when each source was last searched or consulted. |  |
| Search strategy | 7 | Present the full search strategies for all databases, registers and websites, including any filters and limits used. | Supplementary Table 2 |
| Selection process | 8 | Specify the methods used to decide whether a study met the inclusion criteria of the review, including how many reviewers screened each record and each report retrieved, whether they worked independently, and if applicable, details of automation tools used in the process. |  |
| Data collection process | 9 | Specify the methods used to collect data from reports, including how many reviewers collected data from each report, whether they worked independently, any processes for obtaining or confirming data from study investigators, and if applicable, details of automation tools used in the process. |  |
| Data items | 10a | List and define all outcomes for which data were sought. Specify whether all results that were compatible with each outcome domain in each study were sought (e.g. for all measures, time points, analyses), and if not, the methods used to decide which results to collect. |  |
|  | 10b | List and define all other variables for which data were sought (e.g. participant and intervention characteristics, funding sources). Describe any assumptions made about any missing or unclear information. |  |
| Study risk of bias assessment | 11 | Specify the methods used to assess risk of bias in the included studies, including details of the tool(s) used, how many reviewers assessed each study and whether they worked independently, and if applicable, details of automation tools used in the process. |  |
| Effect measures | 12 | Specify for each outcome the effect measure(s) (e.g. risk ratio, mean difference) used in the synthesis or presentation of results. |  |
| Synthesis methods | 13a | Describe the processes used to decide which studies were eligible for each synthesis (e.g. tabulating the study intervention characteristics and comparing against the planned groups for each synthesis (item #5)). |  |
|  | 13b | Describe any methods used to tabulate or visually display results of individual studies and syntheses. |  |
|  | 13c | Describe any methods used to tabulate or visually display results of individual studies and syntheses. |  |
|  | 13d | Describe any methods used to synthesize results and provide a rationale for the choice(s). If meta-analysis was performed, describe the model(s), method(s) to identify the presence and extent of statistical heterogeneity, and software package(s) used. |  |
|  | 13e | Describe any methods used to explore possible causes of heterogeneity among study results (e.g. subgroup analysis, meta-regression). |  |
|  | 13f | Describe any sensitivity analyses conducted to assess robustness of the synthesized results. |  |
| Reporting bias assessment | 14 | Describe any methods used to assess risk of bias due to missing results in a synthesis (arising from reporting biases). |  |
| Certainty assessment | 15 | Describe any methods used to assess certainty (or confidence) in the body of evidence for an outcome. | - |
| **RESULTS** | | |  |
| Study selection | 16a | Describe the results of the search and selection process, from the number of records identified in the search to the number of studies included in the review, ideally using a flow diagram. | Figure 1 |
|  | 16b | Cite studies that might appear to meet the inclusion criteria, but which were excluded, and explain why they were excluded. | Figure 1 |
| Study characteristics | 17 | Cite each included study and present its characteristics. | Table 1 |
| Risk of bias in studies | 18 | Present assessments of risk of bias for each included study. |  |
| Results of individual studies | 19 | For all outcomes, present, for each study: (a) summary statistics for each group (where appropriate) and (b) an effect estimate and its precision (e.g. confidence/credible interval), ideally using structured tables or plots. | Figure 2. |
| Results of syntheses | 20a | For each synthesis, briefly summarize the characteristics and risk of bias among contributing studies. | Figure 2 |
|  | 20b | Present results of all statistical syntheses conducted. If meta-analysis was done, present for each the summary estimate and its precision (e.g. confidence/credible interval) and measures of statistical heterogeneity. If comparing groups, describe the direction of the effect. |  |
|  | 20c | Present results of all investigations of possible causes of heterogeneity among study results. | Supplementary Figure 1-2.  Supplementary Table 3-4. |
|  | 20d | Present results of all sensitivity analyses conducted to assess the robustness of the synthesized results. | Figure 3  Supplementary Table 5 |
| Reporting biases | 21 | Present assessments of risk of bias due to missing results (arising from reporting biases) for each synthesis assessed. | Figure 4 |
| Certainty of evidence | 22 | Present assessments of certainty (or confidence) in the body of evidence for each outcome assessed. | - |
| **DISCUSSION** | | |  |
| Discussion | 23a | Provide a general interpretation of the results in the context of other evidence. |  |
|  | 23b | Discuss any limitations of the evidence included in the review. |  |
|  | 23c | Discuss any limitations of the review processes used. |  |
|  | 23d | Discuss implications of the results for practice, policy, and future research. |  |
| **OTHER INFORMATION** | | |  |
| Registration and protocol | 24a | Provide registration information for the review, including register name and registration number, or state that the review was not registered. |  |
|  | 24b | Indicate where the review protocol can be accessed, or state that a protocol was not prepared. |  |
|  | 24c | Describe and explain any amendments to information provided at registration or in the protocol. |  |
| Support | 25 | Describe sources of financial or non-financial support for the review, and the role of the funders or sponsors in the review. |  |
| Competing interests | 26 | Declare any competing interests of review authors. |  |
| Availability of data, code and other materials | 27 | Report which of the following are publicly available and where they can be found: template data collection forms; data extracted from included studies; data used for all analyses; analytic code; any other materials used in the review. |  |

# Supplementary Table 2 Systematic search detail (record number: 2025.10.31)

| **The search strategy for PubMed** | | |
| --- | --- | --- |
| **Sequence** | **Search terms** | **Hits** |
| #1 | Acute Kidney Injury[MeSH Terms] | 62,183 |
| #2 | ((((((((((((((((((((((Acute Kidney Injuries[Title/Abstract]) OR (Kidney Injuries, Acute[Title/Abstract])) OR (Kidney Injury, Acute[Title/Abstract])) OR (Acute Renal Injury[Title/Abstract])) OR (Acute Renal Injuries[Title/Abstract])) OR (Renal Injuries, Acute[Title/Abstract])) OR (Renal Injury, Acute[Title/Abstract])) OR (Kidney Failure, Acute[Title/Abstract])) OR (Acute Kidney Failures[Title/Abstract])) OR (Kidney Failures, Acute[Title/Abstract])) OR (Acute Kidney Failure[Title/Abstract])) OR (Acute Renal Failure[Title/Abstract])) OR (Acute Renal Failures[Title/Abstract])) OR (Renal Failures, Acute[Title/Abstract])) OR (Renal Failure, Acute[Title/Abstract])) OR (Renal Insufficiency, Acute[Title/Abstract])) OR (Acute Renal Insufficiencies[Title/Abstract])) OR (Renal Insufficiencies, Acute[Title/Abstract])) OR (Acute Kidney Insufficiency[Title/Abstract])) OR (Acute Renal Insufficiency[Title/Abstract])) OR (Kidney Insufficiency, Acute[Title/Abstract])) OR (Acute Kidney Insufficiencies[Title/Abstract])) OR (Kidney Insufficiencies, Acute[Title/Abstract]) | 72,696 |
| #3 | #1 OR #2 | 87,570 |
| #4 | (((((((((((((((((((Critical Illness[Title/Abstract]) OR (Critical Illnesses[Title/Abstract])) OR (Illness, Critical[Title/Abstract])) OR (Illnesses, Critical[Title/Abstract])) OR (Critically Ill[Title/Abstract])) OR (Critical Care[Title/Abstract])) OR (Care, Critical[Title/Abstract])) OR (Intensive Care[Title/Abstract])) OR (Care, Intensive[Title/Abstract])) OR (Surgical Intensive Care[Title/Abstract])) OR (Care, Surgical Intensive[Title/Abstract])) OR (Intensive Care, Surgical[Title/Abstract])) OR (critical patients[Title/Abstract])) OR (critically ill patients[Title/Abstract])) OR (serious illness[Title/Abstract])) OR (seriously ill[Title/Abstract])) OR (critical care medicine[Title/Abstract])) OR (intensive care units[Title/Abstract])) OR (surgical intensive care unit[Title/Abstract])) OR (ICU[Title/Abstract]) | 345,280 |
| #5 | ((((triglyceride glucose index[Title/Abstract]) OR (TyG index[Title/Abstract])) OR (triglyceride-glucose index[Title/Abstract])) OR (TyG[Title/Abstract])) OR (triglyceride glucose[Title/Abstract]) | 3,700 |
| #6 | #3 AND #4 AND #5 | 20 |
| **The search strategy for Web of science** | | |
| **Sequence** | **Search terms** | **Hits** |
| #1 | TS=(Acute Kidney Injury) | 62,726 |
| #2 | ((((AB=(Acute Kidney Injuries)) OR AB=(Kidney Injuries, Acute)) OR AB=(Kidney Injury, Acute)) OR AB=(Acute Renal Injury)) OR AB=(Acute Renal Injuries) | 41,790 |
| #3 | (((((((AB=(Renal Injuries, Acute)) OR AB=(Renal Injury, Acute)) OR AB=(Kidney Failure, Acute)) OR AB=(Acute Kidney Failures)) OR AB=(Kidney Failures, Acute)) OR AB=(Acute Kidney Failure)) OR AB=(Acute Renal Failure)) OR AB=(Acute Renal Failures) | 42,650 |
| #4 | (((((((((AB=(Renal Failures, Acute)) OR AB=(Renal Failure, Acute)) OR AB=(Renal Insufficiency, Acute)) OR AB=(Acute Renal Insufficiencies)) OR AB=(Renal Insufficiencies, Acute)) OR AB=(Acute Kidney Insufficiency)) OR AB=(Acute Renal Insufficiency)) OR AB=(Kidney Insufficiency, Acute)) OR AB=(Acute Kidney Insufficiencies)) OR AB=((Kidney Insufficiencies, Acute) | 20,982 |
| #5 | #1 OR #2 OR #3 OR #4 | 81,180 |
| #6 | (((((((((AB=(Critical Illness)) OR AB=(Critical Illnesses)) OR AB=(Illness, Critical)) OR AB=(Illnesses, Critical)) OR AB=(Critically Ill)) OR AB=(Critical Care)) OR AB=(Care, Critical)) OR AB=(Intensive Care)) OR AB=(Care, Intensive)) OR AB=(Surgical Intensive Care) | 254,295 |
| #7 | (((((((((AB=(Care, Surgical Intensive)) OR AB=(Intensive Care, Surgical)) OR AB=(critical patients)) OR AB=(critically ill patients)) OR AB=(serious illness)) OR AB=(seriously ill)) OR AB=(critical care medicine)) OR AB=(intensive care units)) OR AB=(surgical intensive care unit)) OR AB=(ICU) | 363,993 |
| #8 | #6 OR #7 | 424,220 |
| #9 | ((((AB=(triglyceride glucose index)) OR AB=(TyG index)) OR AB=(triglyceride-glucose index)) OR AB=(TyG)) OR AB=(triglyceride glucose) | 37,350 |
| #10 | #5 AND #8 AND #9 | 33 |
| **The search strategy for EMBASE** | | |
| **Sequence** | **Search terms** | **Hits** |
| #1 | 'acute kidney failure'/exp | 157,208 |
| #2 | 'acute kidney injuries':ab,ti OR 'kidney injuries, acute':ab,ti OR 'kidney injury, acute':ab,ti OR 'acute renal injury':ab,ti OR 'acute renal injuries':ab,ti | 3,207 |
| #3 | 'renal injuries, acute':ab,ti OR 'renal injury, acute':ab,ti OR 'kidney failure, acute':ab,ti OR 'acute kidney failures':ab,ti OR 'kidney failures, acute':ab,ti OR 'acute kidney failure':ab,ti OR 'acute renal failure':ab,ti OR 'acute renal failures':ab,ti | 39,472 |
| #4 | 'renal failures, acute':ab,ti OR 'renal failure, acute':ab,ti OR 'renal insufficiency, acute':ab,ti OR 'acute renal insufficiencies':ab,ti OR 'renal insufficiencies, acute':ab,ti OR 'acute kidney insufficiency':ab,ti OR 'acute renal insufficiency':ab,ti OR 'kidney insufficiency, acute':ab,ti OR 'acute kidney insufficiencies':ab,ti OR 'kidney insufficiencies, acute':ab,ti | 2,871 |
| #5 | #1 OR #2 OR #3 OR #4 | 167,237 |
| #6 | 'critical illness':ab,ti OR 'critical illnesses':ab,ti OR 'illness, critical':ab,ti OR 'critically ill':ab,ti OR 'critical care':ab,ti OR 'care, critical':ab,ti OR 'care, intensive':ab,ti OR 'surgical intensive care':ab,ti | 187,512 |
| #7 | 'care, surgical intensive':ab,ti OR 'intensive care, surgical':ab,ti OR 'critical patients':ab,ti OR 'critically ill patients':ab,ti OR 'serious illness':ab,ti OR 'seriously ill':ab,ti OR 'critical care medicine':ab,ti OR 'intensive care units':ab,ti OR 'surgical intensive care unit':ab,ti OR 'icu':ab,ti | 312,  225 |
| #8 | #6 OR #7 | 387,326 |
| #9 | 'triglyceride glucose index':ab,ti OR 'tyg index':ab,ti OR 'triglyceride-glucose index':ab,ti OR 'tyg':ab,ti OR 'triglyceride glucose':ab,ti | 4,449 |
| #10 | #5 AND #8 AND #9 | 30 |

# Supplementary Table 3 Results of subgroup analysis (OR)

| Subgroup | No. of studies | Odds ratio | 95% confidence interval | *P* value | I^2^ (%) | P value for heterogeneity |
| --- | --- | --- | --- | --- | --- | --- |
| Gender | | | | | | |
| Male | 11 | 1.46 | 1.33-1.60 | <0.00001 | 13 | 0.32 |
| Female | 11 | 1.80 | 1.40-2.31 | <0.00001 | 69 | 0.0004 |
| Age | | | | | | |
| Advanced age | 11 | 1.41 | 1.23-1.62 | <0.00001 | 65 | 0.001 |
| Young age | 11 | 1.54 | 1.31-1.80 | <0.00001 | 65 | 0.001 |
| BMI | | | | | | |
| High BMI | 3 | 1.35 | 0.95-1.90 | 0.09 | 6 | 0.35 |
| Low BMI | 3 | 1.42 | 1.13-1.78 | 0.002 | 21 | 0.28 |
| Race | | | | | | |
| White | 3 | 1.34 | 1.17-1.54 | <0.0001 | 28 | 0.25 |
| Black | 2 | 1.36 | 0.92-2.00 | 0.12 | 66 | 0.08 |
| CKD | | | | | | |
| Yes | 3 | 1.27 | 1.08-1.49 | 0.004 | 14 | 0.31 |
| No | 3 | 1.34 | 1.12-1.60 | 0.001 | 65 | 0.06 |
| Hypertension | | | | | | |
| Yes | 9 | 1.60 | 1.30-1.97 | <0.0001 | 58 | 0.01 |
| No | 9 | 1.54 | 1.25-1.89 | <0.0001 | 72 | 0.0004 |
| DM | | | | | | |
| Yes | 10 | 1.37 | 1.19-1.57 | <0.00001 | 13 | 0.32 |
| No | 10 | 1.56 | 1.31-1.85 | <0.00001 | 64 | 0.003 |
| Atrial fibrillation | | | | | | |
| Yes | 2 | 1.45 | 0.92-2.29 | 0.11 | 56 | 0.13 |
| No | 2 | 1.68 | 0.95-2.98 | 0.07 | 93 | 0.0001 |
| Study design | | | | | | |
| Cross-sectional study | 3 | 1.75 | 1.15-2.67 | 0.009 | 87 | 0.0005 |
| Cohort study | 9 | 1.33 | 1.13-1.58 | 0.0009 | 86 | <0.00001 |

# Supplementary Table 4 Results of subgroup analysis (HR)

| Subgroup | No. of studies | Hazard ratio | 95% confidence interval | *P* value | I^2^ (%) | P value for heterogeneity |
| --- | --- | --- | --- | --- | --- | --- |
| Gender | | | | | | |
| Male | 6 | 1.35 | 1.16-1.59 | 0.0002 | 88 | <0.00001 |
| Female | 6 | 1.39 | 1.20-1.62 | <0.0001 | 68 | 0.004 |
| Age | | | | | | |
| Advanced age | 5 | 1.52 | 1.23-1.88 | 0.0001 | 85 | <0.0001 |
| Young age | 5 | 1.51 | 1.31-1.75 | <0.00001 | 59 | 0.04 |
| BMI | | | | | | |
| High BMI | 5 | 1.45 | 1.05-2.01 | 0.02 | 88 | <0.00001 |
| Low BMI | 5 | 1.40 | 1.06-1.84 | 0.02 | 52 | 0.08 |
| CKD | | | | | | |
| Yes | 5 | 1.29 | 1.16-1.45 | <0.00001 | 42 | 0.14 |
| No | 5 | 1.45 | 1.26-1.66 | <0.00001 | 75 | 0.003 |
| Hypertension | | | | | | |
| Yes | 5 | 1.17 | 1.09-1.25 | <0.00001 | 37 | 0.17 |
| No | 5 | 1.39 | 1.16-1.66 | 0.0003 | 86 | <0.0001 |
| DM | | | | | | |
| Yes | 6 | 1.27 | 1.07-1.50 | 0.007 | 79 | 0.0002 |
| No | 6 | 1.46 | 1.17-1.81 | 0.0008 | 93 | <0.00001 |
| Congestive heart failure | | | | | | |
| Yes | 2 | 1.18 | 1.06-1.31 | 0.002 | 13 | 0.28 |
| No | 2 | 1.14 | 1.04-1.26 | 0.007 | 62 | 0.11 |
| Acute myocardial infarction | | | | | | |
| Yes | 2 | 1.41 | 1.18-1.69 | 0.0002 | 0 | 0.85 |
| No | 2 | 1.60 | 1.47-1.73 | <0.00001 | 0 | 0.91 |

# Supplementary Table 5 Sensitivity analysis

| Outcome | Omitted Study | Data with study removed OR/HR (95% CI) | *P* | *I^2^* |
| --- | --- | --- | --- | --- |
| AKI (OR) | Cai DB 2024 | 1.38 [1.21, 1.57] | <0.00001 | 92% |
|  | Fang YJ 2024 | 1.36 [1.19, 1.54] | <0.00001 | 93% |
|  | Hou B 2025 | 1.44 [1.27, 1.64] | <0.00001 | 72% |
|  | Huang J 2024 | 1.35 [1.19, 1.53] | <0.00001 | 92% |
|  | Jin ZH 2023 | 1.43 [1.21, 1.71] | <0.0001 | 87% |
|  | Jin ZH 2024 | 1.34 [1.18, 1.52] | <0.00001 | 92% |
|  | Lu Y 2025 | 1.41 [1.23, 1.62] | <0.00001 | 93% |
|  | Pan RJ 2025 | 1.39 [1.22, 1.58] | <0.00001 | 93% |
|  | Qiu XY 2025 | 1.35 [1.19, 1.54] | <0.00001 | 92% |
|  | Wang X 2024 | 1.38 [1.21, 1.58] | <0.00001 | 93% |
|  | Zhang F 2025 | 1.40 [1.23, 1.60] | <0.00001 | 93% |
|  | Zhang Z 2025 | 1.44 [1.26, 1.65] | <0.00001 | 93% |
| AKI (HR) | Shi Y 2024 | 1.43 [1.11, 1.84] | 0.006 | 90% |
|  | Wang Z 2025 | 1.40 [1.10, 1.78] | 0.007 | 90% |
|  | Yang ZW 2023 | 1.40 [1.09, 1.80] | 0.009 | 89% |
|  | Zhang PR 2025 | 1.55 [1.37, 1.76] | <0.00001 | 23% |
|  | Zhang WB 2024 | 1.34 [1.10, 1.63] | 0.003 | 75% |
|  | Zhang Y 2025 | 1.46 [1.11, 1.91] | 0.007 | 90% |

Supplementary Fig. 1 Subgroup analysis of AKI (OR)

Gender: (A) Male, (B) Female; Age: (C) Advanced age, (D) Young age; Race: (E) White, (F) Black; CKD: (G) With CKD; (H) Without CKD; Hypertension: (I) With hypertension, (J) Without hypertension; DM: (K) With DM, (L) Without DM; Atrial fibrillation: (M) With atrial fibrillation, (N) Without Atrial fibrillation; Study design: (O) cross-sectional study, (P) cohort study; BMI: (Q) High BMI, (R) Low BMI; .


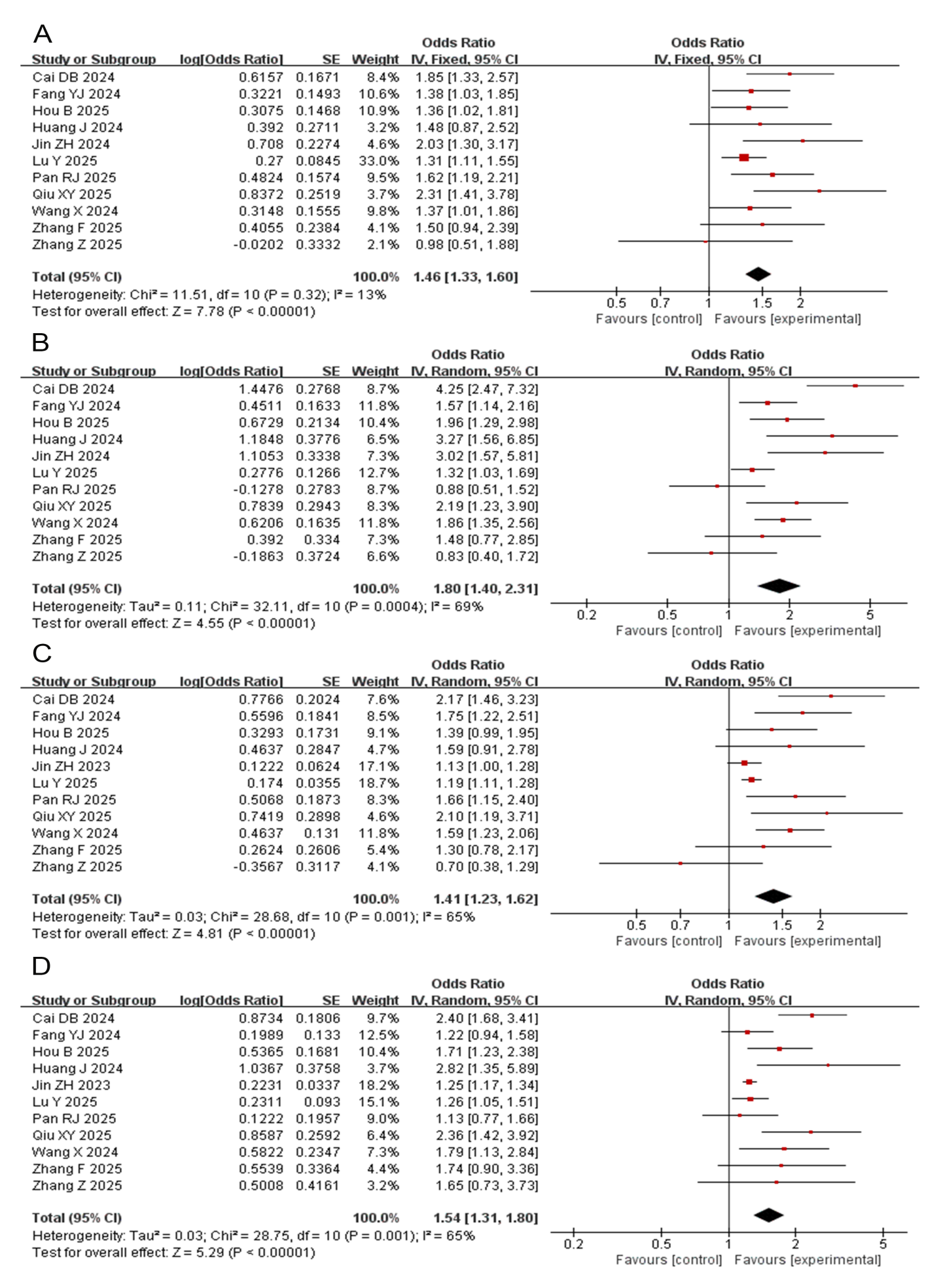


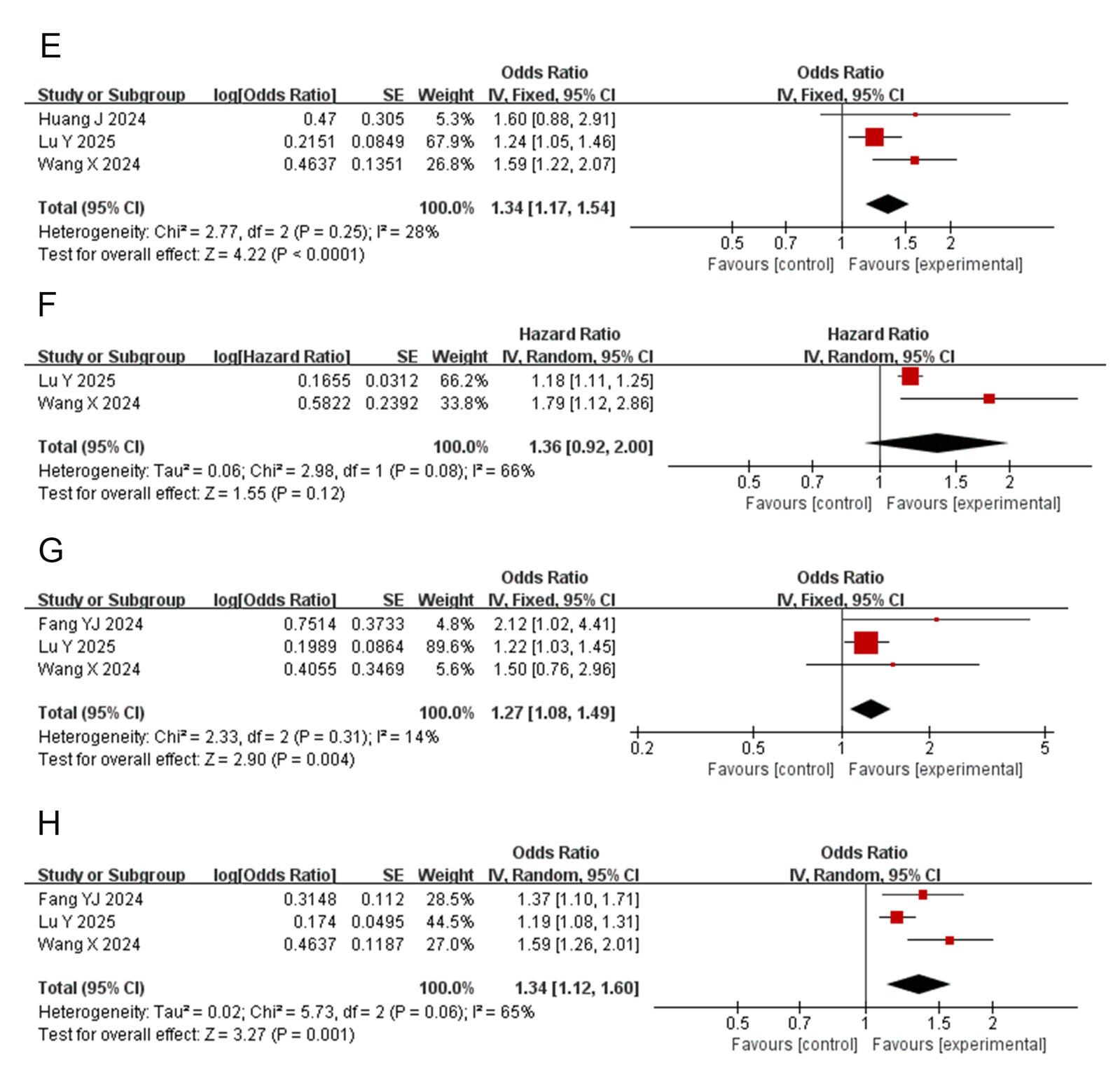


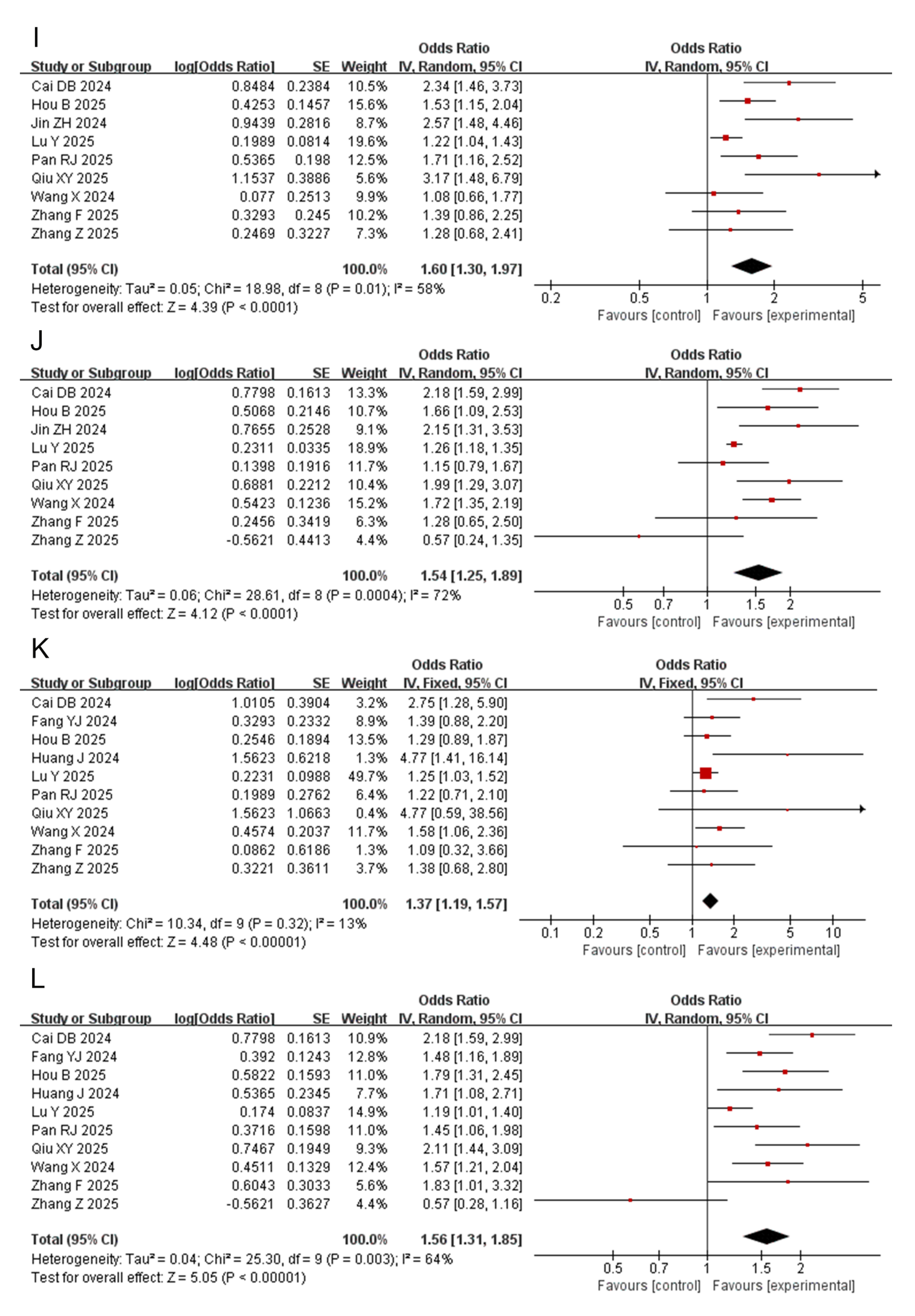


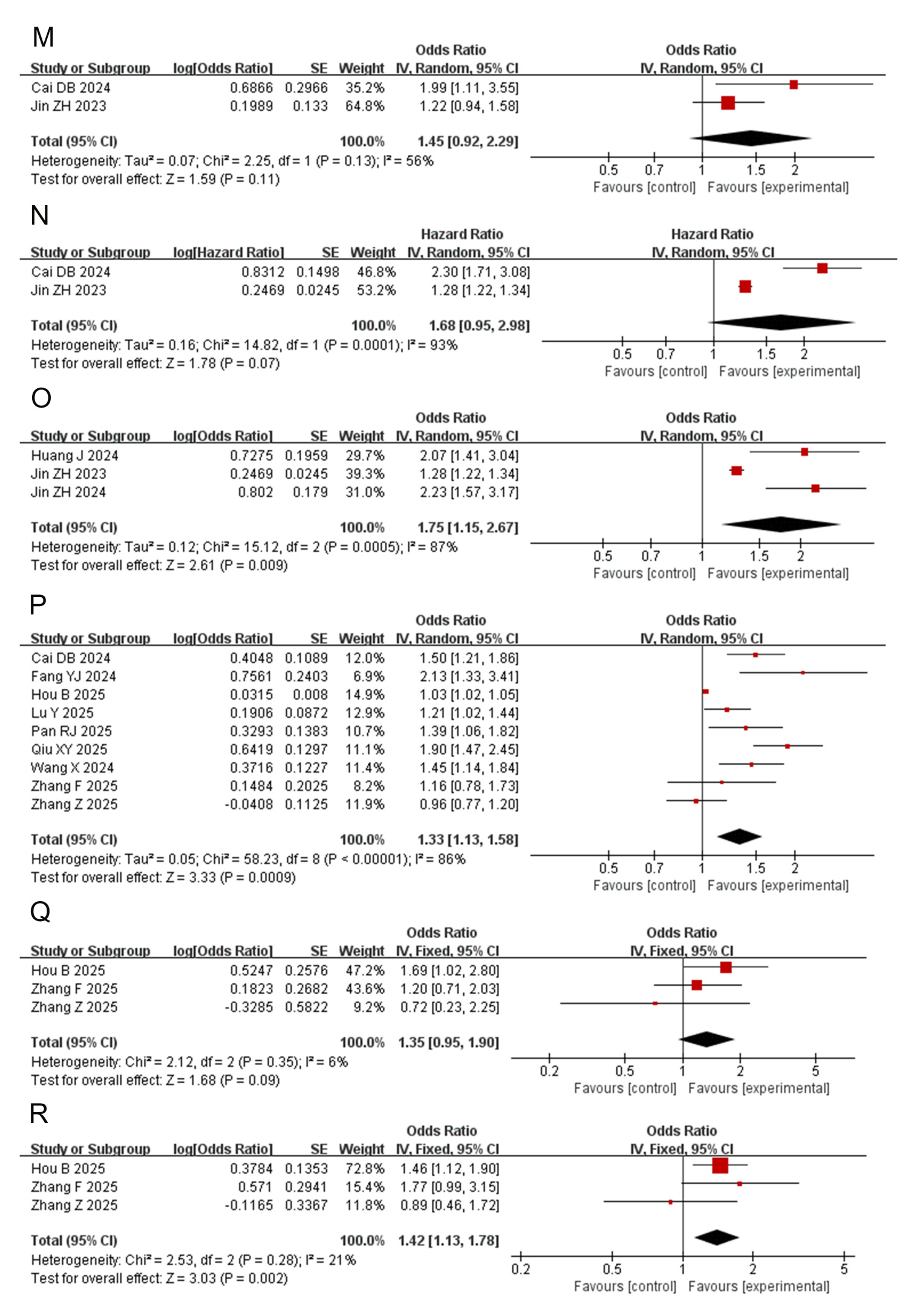


Supplementary Fig. 2 Subgroup analysis of AKI (HR)

Gender: (A) Male, (B) Female; Age: (C) Advanced age, (D) Young age; Race: BMI: (E) High BMI, (F) Low BMI; CKD: (G) With CKD; (H) Without CKD; Hypertension: (I) With hypertension, (J) Without hypertension; DM: (K) With DM, (L) Without DM; Congestive heart failure: (M) With congestive heart failure, (N) Without congestive heart failure; Acute myocardial infarction: (O) With acute myocardial infarction, (P) Without acute myocardial infarction.


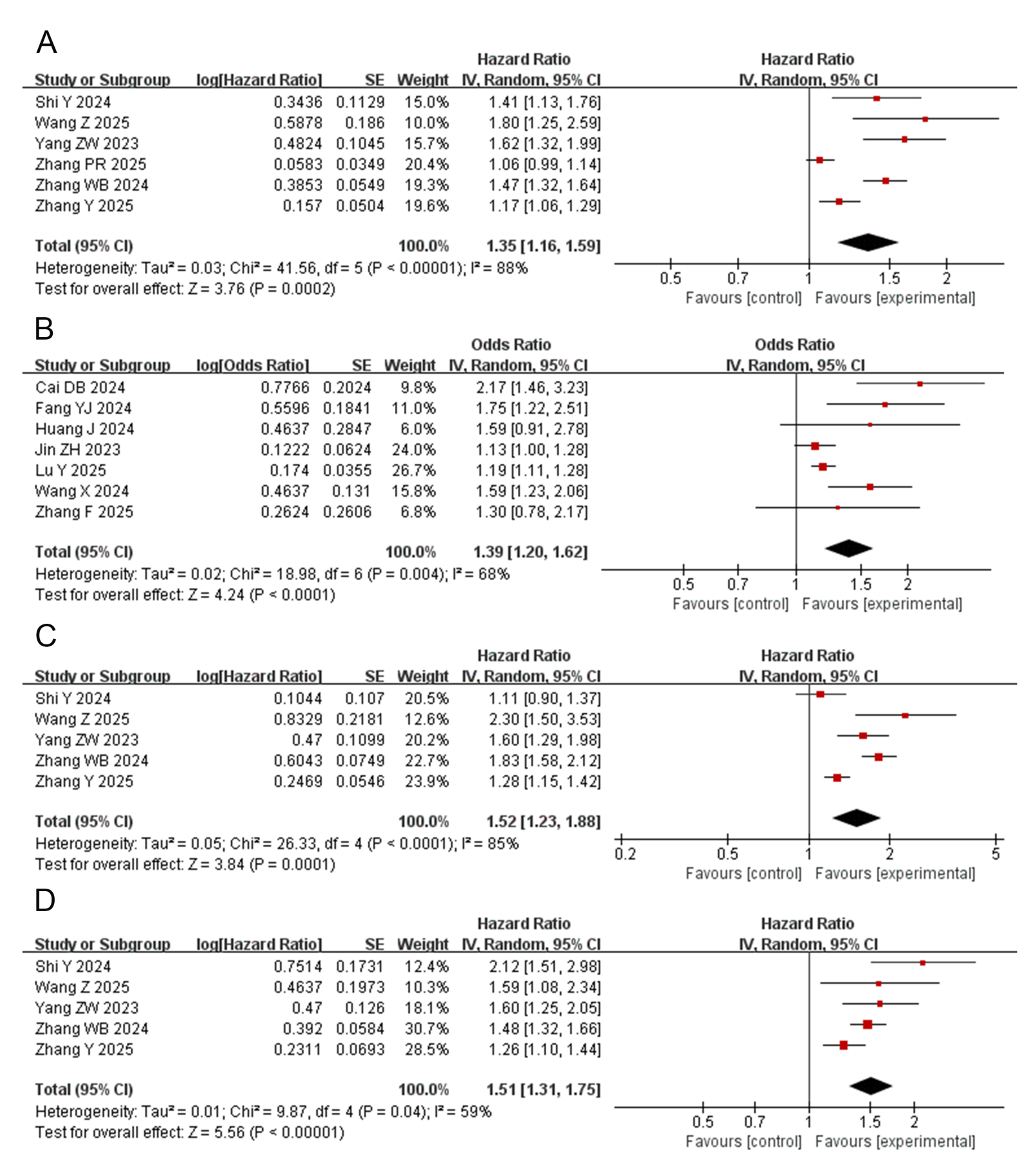


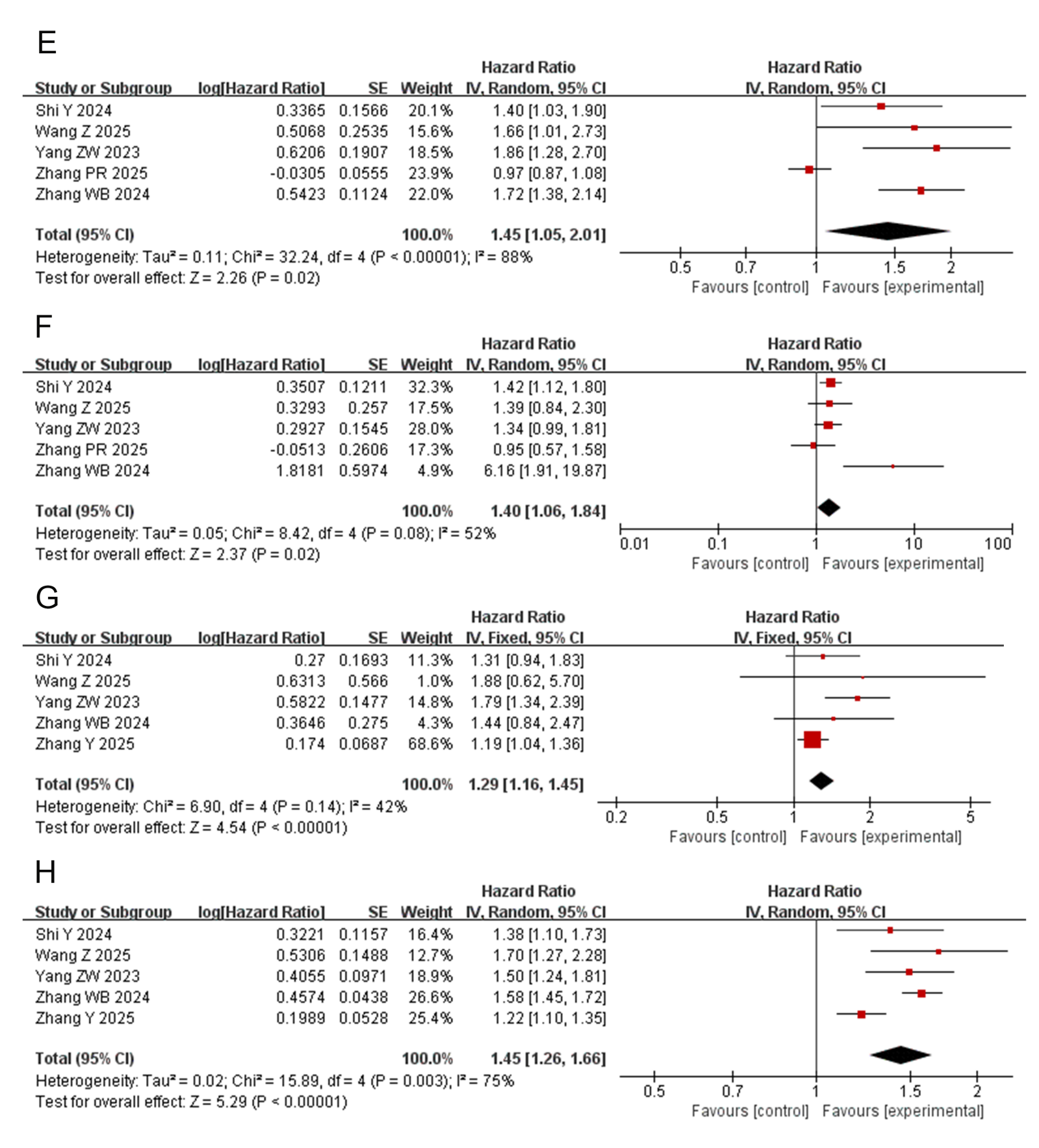


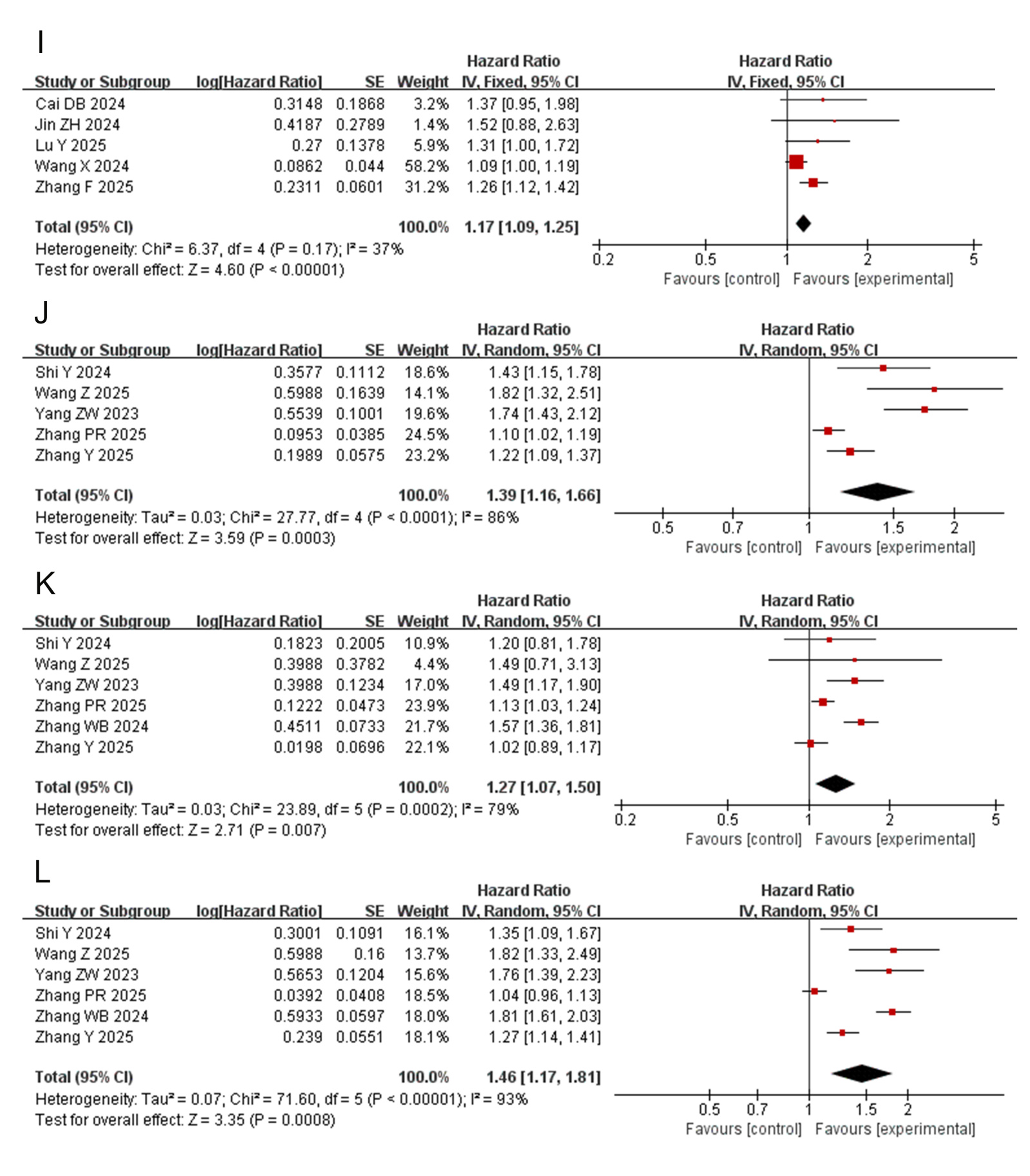


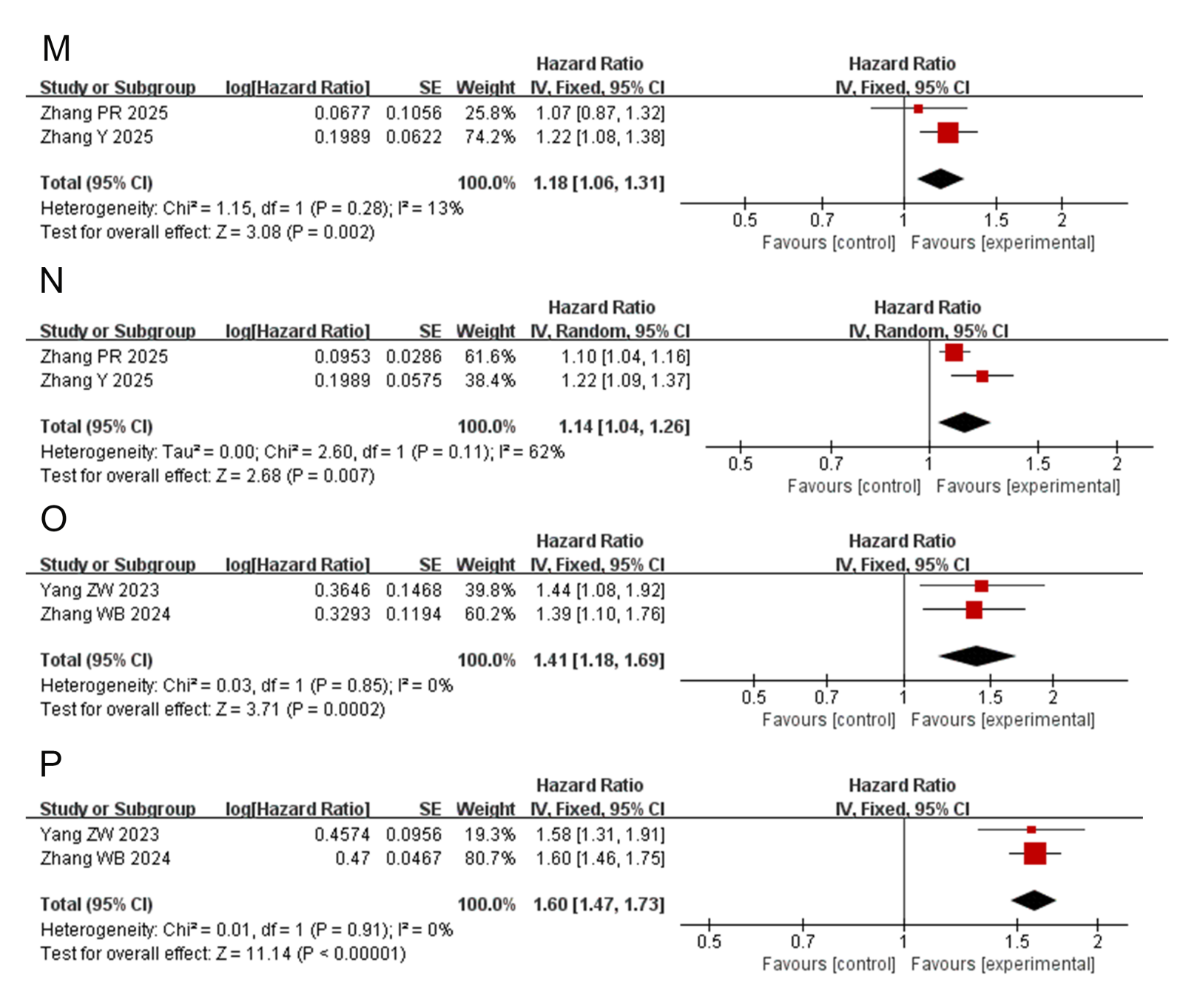

Supplement: Supplementary file 1 [file DataSheet1.docx]
